# DO WELFARE RECEIPTS CROWD OUT PRIVATE TRANSFERS FROM FAMILIES AND FRIENDS? EVIDENCE FROM PANEL DATA IN CHINA

**DOI:** 10.1093/geroni/igz038.1231

**Published:** 2019-11-08

**Authors:** Yalu Zhang

**Affiliations:** Columbia University, New York, New York, United States

## Abstract

Many countries are undergoing an unprecedented challenge to provide financial support to the older generation and guarantee their livelihood and wellbeing. China is no exception. Rural older adults in China have been becoming even more vulnerable to lack of care and inadequate financial resources as the growth of urbanization and labor migration has intensified. Therefore, it becomes increasingly difficult to follow the traditional family support model for the aged. Using the panel data of the China Health and Retirement Longitudinal Study 2011, 2013, and 2015 and a system generalized methods of moment (GMM), this paper examined the dynamic relationship between welfare receipts and monetary transfers from families and friends among rural and urban older adults (n=9,496) in China. The results show that the welfare receipts do not induce any “crowd-out” or “crowd-in” effects on rural older adults’ private transfer receipts. The incidence and amount of private transfers that occurred among rural older adults are more likely to be determined by the private transfers they received in prior waves. The intensity of catastrophic health expenditure itself does not affect the occurrence and size of private transfers. This study, on the one hand, confirms that among rural recipients, public transfers do not substitute private transfers, which most of the policymakers have long been concerned about. However, on the other hand, it also reveals the shortcoming of current public transfer policies—the generosity of public transfers does not enable rural older adults to be financially independent of intra-family transfers.

